# Contrasting effect of recombinant human erythropoietin on breast cancer cell response to cisplatin induced cytotoxicity

**DOI:** 10.2478/v10019-012-0037-8

**Published:** 2012-06-19

**Authors:** Nina Trost, Peter Juvan, Gregor Sersa, Natasa Debeljak

**Affiliations:** 1 Center for Functional Genomics and Bio-chips, Institute of Biochemistry, Faculty of Medicine, University of Ljubljana, Slovenia; 2 Institute of Oncology, Department of Experimental Oncology, Ljubljana, Slovenia; 3 Medical Center for Molecular Biology, Institute of Biochemistry, Faculty of Medicine, University of Ljubljana, Slovenia

**Keywords:** breast cancer, erythropoietin, cisplatin, cytotoxicity

## Abstract

**Background:**

Human recombinant erythropoietin (rHuEpo) that is used for the treatment of the chemotherapy-induced anaemia in cancer patients was shown to cause detrimental effects on the course of disease due to increased adverse events inflicting patient’s survival, potentially related to rHuEpo-induced cancer progression. In this study, we elucidate the effect of rHuEpo administration on breast cancer cell proliferation and gene expression after cisplatin (cDDP) induced cytotoxicity.

**Materials and methods:**

Two breast carcinoma models, MCF-7 and MDA-MB-231 cell lines, were used differing in oestrogen (ER) and progesterone (PR) receptors and *p53* status. Cells were cultured with or without rHuEpo for 24 h or 9 weeks and their growth characteristics after cDDP treatment were assessed together with expression of genes involved in the *p53*-signaling pathway.

**Results:**

Short-term exposure of breast cancer cells to rHuEpo lowers their proliferation and reduces cDDP cytotoxic potency. In contrast, long-term exposure of MCF-7 cells to rHuEpo increases proliferation and predisposes MCF-7 cells to cDDP cytotoxicity, but has no effect on MDA-MB-231 cells. MDA-MB-231 cells show altered level of ERK phosphorylation, indicating involvement of MAPK signalling pathway. Gene expression analysis of *p53*-dependent genes and *bcl-2* gene family members confirmed differences between long and short-term rHuEpo effects, indicating the most prominent changes in *BCL2* and *BAD* expression.

**Conclusions:**

Proliferation and survival characteristics of MCF-7 cells are reversely modulated by the length of the rHuEpo exposure. On the other hand, MDA-MB-231 cells are almost irresponsive to long-term rHuEpo, supposedly due to the mutated *p53* and ER(+)/PR(−) status. The p53 and ER/PR status may predict tumour response on rHuEpo and cDDP treatment.

## Introduction

Erythropoietin (Epo) is a hormone of renal origin that upon its binding to the cognate erythropoietin receptor (EpoR) acts as one of the main regulators of proliferation and differentiation of erythroid progenitors in bone marrow.^1^ EpoR expression is not limited only to erythropoietic cells but it is expressed also in a wide variety of non-hematopoietic cells.^1^–^3^ Epo is the only hematopoietic growth factor whose expression is regulated by tissue hypoxia.^4^ Accumulating evidence has proven that Epo exerts additional tissue-protective effects for multiple tissues, for example in ischemic and degenerative heart and brain diseases.^5^ Expression of EpoR was detected in tumour tissues and question has arisen whether Epo promotes tumour cell survival and stimulates tumour growth. Direct influences on normal and tumour cell progression therefore require the presence of functional surface EpoR to trigger downstream signalling pathways, namely JAK/STAT5, PI3K/Akt, Ras/MAPK and PKC.^6^,^7^ It was suggested that Epo may exert the pleiotropic mode of action.^8^

Severe anaemia is a frequent side effect of cancer chemotherapy, resulting mainly from chemotherapy induced inhibition of erythroid cell maturation in the bone marrow and interference with the ability of kidney to produce Epo. Onset of anaemia is associated with reduced quality-of-life and is thought to concur with the development of more aggressive cancer phenotypes due to lowered tumour oxygenation.^9^ In the early days, rHuEpo was shown to be a safe and effective treatment of choice, improving quality-of-life and reducing the need for blood transfusions.^10^,^11^ However, data from clinical trials in head and neck (ENHANCE)^12^ and breast cancer patients (BEST)^13^,^14^ and from subsequent meta-analyses (*e.g*., EPO-CAN 20, GOG 191 and trials with breast cancer)^15^–^18^ gave conflicting results indicating that rHuEpo treatment is reducing progression-free and overall survival with increasing haemoglobin level over 120 g/L.^19^ Breast cancer is the most common cancer among women in the world and as such represents an important health care challenge.^20^ Cisplatin (cDDP), a very potent anti-tumour agent, is used for the therapy of several malignancies.^21^–^23^ It shows high activity as first-line chemotherapy in advanced breast cancer.^24^ The formation of DNA-cDDP adducts translate cDDP-induced DNA damage to inhibition of DNA synthesis, suppression of RNA transcription causing cell cycle arrest that finally culminates in the activation of apoptosis.^25^ Apoptosis is one of the pathways of programmed cell death that is markedly influenced by the variety of genes, among which the most important are the tumour-suppressor gene *p53* and members of the *bcl-2* gene family. Mutations in *p53* have been shown to confer sensitivity to drugs whose toxicity is modulated by nuclear excision repair, such as ERCC1.^26^ The main drawback of cDDP based chemotherapy is the occurrence of resistance.^27^

In this study we focused on MCF-7 and MDAMB-231 breast cancer cell lines in order to address potential effect of Epo on the response of tumour cells to the cDDP cytotoxicity. rHuEpo was reported to stimulate the proliferation of several human breast cancer cell lines that were expressing functional EpoR^28^, including both cell lines used in this study. There are several well established genetic differences between the selected cell lines potentially contributing to cell sensitivity to rHuEpo and cDDP. MCF-7 is oestrogen (ER) and progesterone receptor (PR) positive cell line with wild-type *p53*, while MDA-MB-231 cell line is ER-positive but PR-negative with mutated *p53*. Normal p53 function was shown to have positive implications in the propagation of apoptotic cell death. In line with this, ER(+)/PR(−) breast tumours have more aggressive phenotypes and are less sensitive to growth factor deprivation compared to ER(+)/PR(+).^29^ Moreover, strong correlations between high *EPOR, ER* and *PR* expression were reported and a specific functional association between EpoR and ERα was postulated.^30^ Similar studies were performed with different cell types, namely renal carcinoma cells, melanoma, malignant glioma, cervical cancer cells and mesothelioma cells^31^–^34^, reporting contradictory effects of Epo on cell survival after cDDP treatment. However this is the first study focusing on the effects of rHuEpo and cDDP in breast cancer with described genotype (p53, ER/PR status). With cell proliferation, viability and clonogenic assays we evaluated short (24 h, 12 days) and long-term (9 weeks) effect of rHuEpo treatment on MCF-7 and MDA-MB-231 growth characteristics, their sensitivity to cDDP and potential synergism between both treatments. Genes involved in the process of cell apoptosis, specifically those included in the *p53*-signaling pathway and the *bcl-2* gene family, because they mediate majority of cytotoxic stimuli, were analysed with qPCR. Using western blot, we analysed the phosphorylation status of extracellular signal-regulated kinase (ERK, MAPK), protein kinase B (Akt, PI-3K) and signal transducers and activators of transcription 5 (STAT5, Jak/STAT5) proteins that are thought to be activated upon rHuEpo treatment^6^,^7^,^35^ or were previously shown to be crucial for cDDP induced apoptotic response.^36^

## Materials and methods

### Cell lines and cell culture pretreatments

MCF-7 and MDA-MB-231 human breast epithelial cells and UT7/Epo human leukemic, an Epo dependent cell line, were maintained in cell culture at 37°C in a humidified 5% (v/v) CO2 atmosphere. MCF-7 and MDA-MB-231 cells were obtained from American Type Culture Collection (ATCC, USA) and were cultured according to the ATCC recommendations. MCF-7 and MDA-MB-231 cells were pretreated with the rHuEpo for 9 weeks (5 and 25 U/mL, Neorecormon, Roche, Germany). In parallel, control cells were cultured in the same conditions, but without rHuEpo. For cell proliferation and cell viability studies, insulin was omitted from the media. cDDP (Pliva, Croatia) was used for cytotoxicity studies (0–200 μM). UT7/Epo cells were kindly provided by C. Lacout (Institute of Cancerology Gustave Roussy, France) and were cultured in alphaMEM medium (Sigma, USA), supplemented with 10% FBS and 2 U/mL rHuEpo and were used as a positive control in western blot analysis.

### Proliferation assays

**FIGURE 1:**
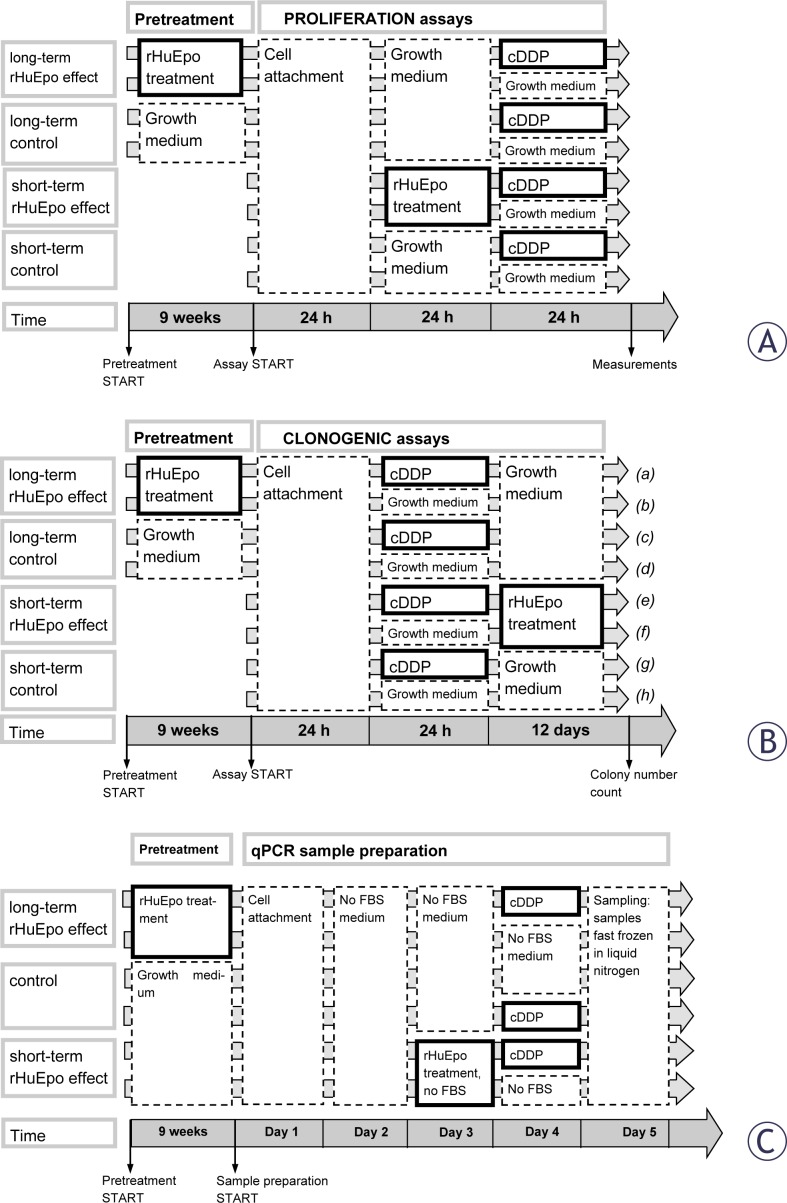
Schematic representation of treatment protocols: (A) proliferation, (B) clonogenic, (C) qPCR and western blot assays.

Cell proliferation assays were performed with colorimetric WST-1 reagent (Roche) on 9 weeks rHuEpo pretreated and 24 h treated MCF-7 and MDA-MB-231 cell lines in parallel with control cells that were cultured without rHuEpo. Cells were exposed to cDDP and cell proliferation was assessed as shown in Figure 1A. 4×10^3^ cells per well were seeded in five-plicates on a 96-well plate and left to adhere in the medium. After two days in culture, cells were exposed to varying concentrations of cDDP (0, 1, 3, 10, 30, 60, 100, 120, 150, 180, 200 μM) for 24 h. Cell proliferation was normalized to the proliferation of control cells that were not exposed to cDDP. All experiments were performed three times.

### Clonogenic assays (CFAs)

Assay was performed on 9 weeks rHuEpo pre-treated and 12 days rHuEpo treated MCF-7 and MDA-MB-231 cells in parallel with control cells that were cultured without rHuEpo. Cells were seeded on 6-well plates at a concentration of 100 cells per well and cultured for 14 days. To address rHuEpo effect, pretreated cells and their controls were cultured in the growth medium without rHuEpo for 14 days (Figure 1B, treatments *b, d, f, h*). rHuEpo and cDDP interaction was evaluated on cells that were exposed to cDDP for 24 h as shown in Figure 1B. Cells were exposed to varying cDDP concentrations (0.01, 0.05, 0.1, 0.5, 1, 6, 10, 12, 18, 20 μM). The medium was changed every 5 days. Colony quantification was done manually and using UviPro analysis system (Uvitec, UK) after crystal violet staining (0.5 %). Colonies were classified as small if containing <100 cells or big otherwise. Surviving fraction of cDDP exposed cells were normalized relative to surviving fraction of non-exposed cells.^25^ Experiments were repeated three times in tri-plicates.

### Gene expression analysis

*Sample preparation.* On day 1, control and rHuEpo pretreated MCF-7 and MDA-MB-231 cells were seeded on 6-well plates in 4 replicates at density 2×10^5^ cells per well (Figure 1C). Medium was changed to serum free medium after 24 h of incubation. Cells were treated with rHuEpo for 24 h on day 3 in order to assess its short-term effect. cDDP treatment was performed on day 4. 10 μM cDDP was used for the treatment of MCF-7 cells and 60 μM for MDA-MB-231 cells. Samples were fast frozen in liquid nitrogen on day 5. RNA was extracted using TRI Reagent (Sigma) and treated with the DNase I (Roche) according to the manufacturer’s instructions. The quality of RNA samples was determined using Agilent bio-analyser (Agilent Technologies, USA) assuring all RNA integrity numbers (RIN) were above 9.8. 1 μg of total RNA was transcribed to cDNA using SuperScript III reverse transcriptase (Invitrogen, USA) according to the manufacturer’s instructions.

*Primer design and qPCR.* Forward and reverse primers were designed to span intron-exon junctions using PrimerExpress software (Applied Biosystems, USA) and their specificity was checked using BLAST algorithm. Primer validation was performed by analysing slope of the standard curve and the presence of a single peak in the melting curve after qPCR analysis. From the cohort of 7 reference genes (Table 1) two most stable (*Rplp0, GAPDH*) were selected for normalization using GeNorm algorithm.^37^ Expression of 13 genes of interest (Table 1) and two selected normalization genes was analysed using SybrGreen chemistry. qPCR was performed on a 384-well platform using LightCycler 480 Real-Time PCR System (Roche). Amplification of specific PCR products was performed in triplicates in a total reaction mixture of 5 μL containing 0.75 μL of cDNA template. Gene expression normalization factors were calculated for each sample based on geometric means of the selected normalization genes.^37^ Minimum Information for Publication of Quantitative Real-Time PCR Experiments (MIQE) guidelines were followed in the performance and interpretation of the qPCR reactions.^38^

### Western blot analysis

Expression of ERK, Akt and STAT5 proteins and their phosphorylated forms was determined by western blotting in the cell lysates of MCF-7 and MDA-MB-231 cells after rHuEpo treatment and exposure to cDDP. 24 h rHuEpo treated and 9 weeks pretreated cells were together with non-treated cells seeded on 6-well plates in the concentration of 1×10^5^ cells per well and left in culture for 48 h. 24 h before treatments, cells were switched to serum free medium. To assess rHuEpo effect, cells were treated with 5 or 25 U/mL rHuEpo for 15 minutes (similarly as shown in Figure 1C except that rHuEpo treatment was applied instead of cDDP). After treatment, the culture medium was aspirated and samples were fast frozen in liquid nitrogen. To assess rHuEpo and cDDP interaction, cells were exposed to two different concentrations of cDDP for 4 h: 30 and 60 μM for MCF-7 cell line and 60 and 120 μM for MDA-MB-231 (similarly as in Figure 3 except for a shorter cDDP) and fast frozen in liquid nitrogen after culture medium was aspirated.

After treatments, cells were lysed for 10 minutes on ice in lysis buffer as described in Kutuk *et al.*^39^ and soluble proteins were recovered in the supernatant following 10 min centrifugation (12000 rpm). Samples of insulin treated (10 μg/mL for 15 min) MCF-7 cells and rHuEpo treated (1 U/mL for 15 min) UT7/Epo cells were used as positive controls. Equal amounts of proteins (10 μg) from each sample were loaded per well. After SDS electrophoresis, proteins were transferred to polyvinylidene difluoride (PVDF) membranes (Immobilon P, Millipore, USA). Membranes were blocked in a blocking solution (5% BSA in 1 mM PBS, 1% Tween-20) for 1 h and incubated in one of the following antibodies and dilutions: anti-ERK (1:1000), anti-Akt (1:600), anti-STAT5 (1:600), anti-P-ERK (1:1000), anti-P-Akt (1:600) and anti-P-STAT5 (1:600). All antibodies were purchased from Cell Signalling Technology and were raised against synthetic peptides in rabbits. Mouse anti-actin antibodies (1:5000, Sigma) were used for loading controls. As a secondary antibody, peroxidase-conjugated anti-rabbit-IgG (1:5000, Sigma) or anti-mouse-IgG (1:5000, Sigma) was used and visualized by chemiluminescence reagent (Pierce ECL Western Blotting Substrate, Thermo Scientific, USA) with CCD camera (FujiFilm, Japan). Membranes were densitometrically analysed using ImageJ software (National Institutes of Health, US)^40^ and ratios between phosphorylated proteins to their non-phosphorylated forms were calculated and compared between samples. All experiments were done in duplicates and repeated twice.

### Statistical analysis

Statistical analysis of the data was performed using Limma package^41^ from Bioconductor analysis tools for R programing language.^42^ The effect of EPO treatment, exposure to cDDP and their interaction in cell survival/proliferation assays, western blot and qPCR was assessed by two-way analysis of variance (ANOVA). Multiple-testing correction using false discovery rate (FDR)^43^ was employed and P<0.05 was considered as statistically significant.

## Results

### Cell proliferation and survival

**FIGURE 2:**
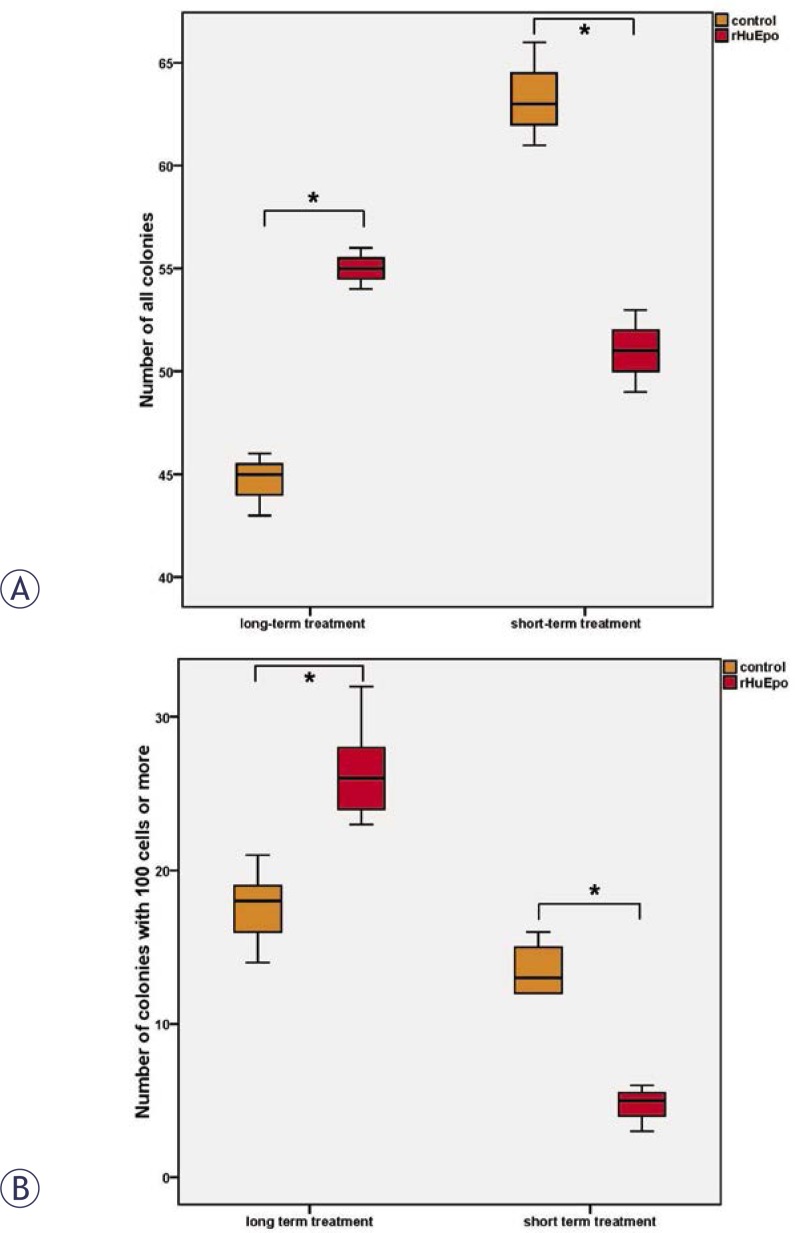
Clonogenic assay with short and long-term treated and non-treated MCF-7 cells: (A) Number of all colonies; (B) Number of colonies with 100 cells or more. Asterisk (*) denotes statistical significant differences for Type I error α = 0.05.

*rHuEpo effect.* Clonogenic assays showed decreased colony number (p = 0.043) together with a drop in colony size (p = 0.0007) in short-term rHuEpo treated MCF-7 cells (12 days) (Figure 2A and B, short-term), indicating a cytotoxic effect and decreased cell proliferation. Contrary in rHuEpo pretreated MCF-7 cells colony number (p = 0.002) and colony size (p = 0.022) were increased (Figure 2A and B, long-term), indicating a positive effect on cell proliferation and survival. In MDA-MB-231 cell line, no significant rHuEpo effect was observed (data not shown).

### Establishment of cDDP inhibitory concentrations that reduced cell survival to 50% (IC50)

The following IC50 concentrations were established for colorimetric assays: 10–30 μM for MCF-7 cell line^44^ and 60–100 μM for MDA-MB-231; and clonogenic assays: 0.1–0.5 μM for MCF-7 cell line and 6–10 μM for MDA-MB-231.

**FIGURE 3:**
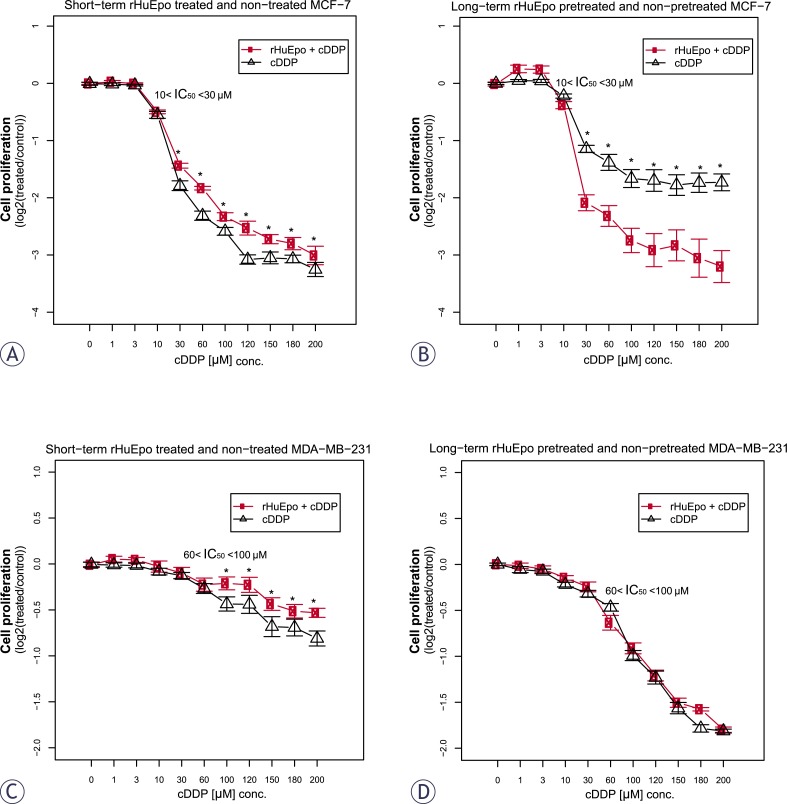
Cell proliferation of short (red line, A and C) and long-term (red line, B and D) rHuEpo treated and non-treated cells (black line) after exposure to cDDP, normalized with the proliferation of control cells that were not exposed to cDDP: (A and B) MCF-7; (C and D) MDA-MB-231 cell line. Asterisk (*) denotes statistical significant differences for Type I error α = 0.05.

*rHuEpo and cDDP interaction.* Colorimetric WST-1 assays revealed protective effect of short-term rHuEpo treatment for MCF-7 and MDA-MB-231 cells that were exposed to cDDP induced cytotoxicity (Figures 3A and 3C, respectively). Contrary, long-term exposure of cells to rHuEpo sensitized MCF-7 cells to cDDP cytotoxicity but had no effect for the MDA-MB-231 cells (Figures 3B and 3D, respectively). This indicates that the time of Epo exposure is crucial for cell response to cDDP treatment.

**FIGURE 4:**
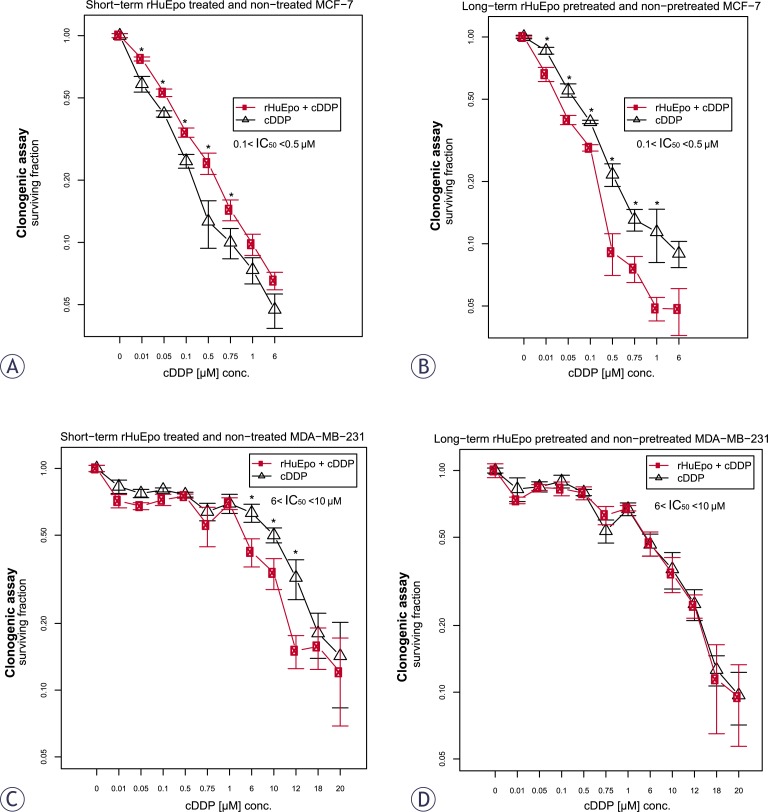
Surviving fraction of short (red line, A and C) and long-term (red line, B and D) rHuEpo treated cells after exposure to cDDP: (A and B) MCF-7; (C and D) MDA-MB-231 cell line. Asterisk (*) denotes statistical significant differences for Type I error α = 0.05.

Clonogenic assays confirmed protective effect of short-term rHuEpo treatment for the MCF-7 cell response to cDDP cytotoxicity (Figure 4A), while they exposed sensitizing effect for MDA-MB-231 cells (Figure 4C). Long term exposure of cells to EPO predisposed MCF-7 cells to CDDP cytotoxicity (Figure 4B) but not the MDA-MB-231 cells (Figure 4D), as shown by WST-1.

### Expression of p53-dependent genes and bcl2-gene family mambers

Expression of 13 genes was measured on control, short-term rHuEpo treated and pretreated MCF-7 and MDA-MB-231 cells that were either exposed to cDDP or not (Figure 1C). qPCR confirmed low *EPOR* expression in all experimental settings with Cq values below 34, a value which was chosen as a cut-off point. *EPOR* expression is therefore not influenced by either increasing confluence of cell cultures or exposure to rHuEpo. Similarly, *CASP3* was not expressed in MCF-7 cells, which is in agreement with Henkels *et al.*^45^

**FIGURE 5:**
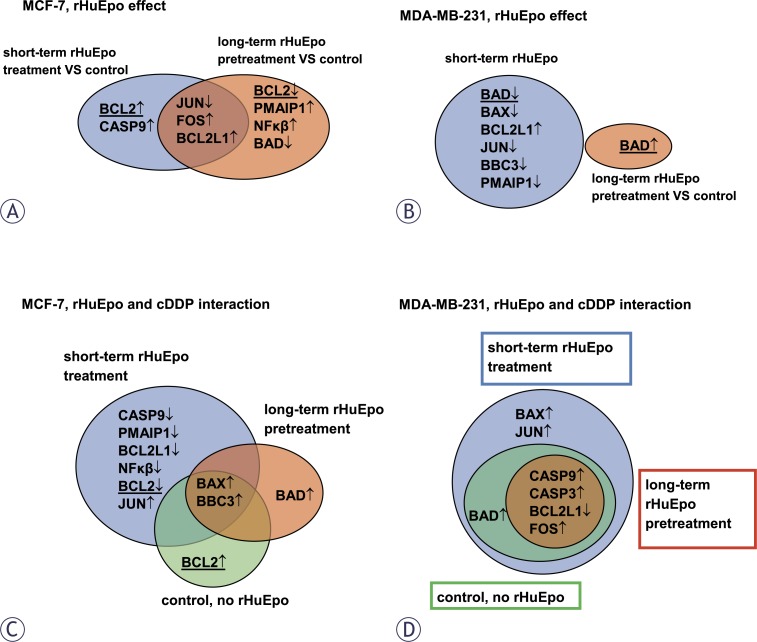
(A and B) Venn’ diagrams representing differential gene expression at different rHuEpo treatments when compared to non-treated cells: (A) MCF-7; (B) MDA-MB-231 cell line. (C and D) Venn’ diagrams representing differential gene expression in cells that were or were not exposed to cDDP at different rHuEpo treatments: (C) MCF-7; (D) MDA-MB-231 cell line. ↑: up-regulation. ↓: down-regulation. Underlined = genes with non-matching direction of change.

*rHuEpo effect.* Venn diagrams on Figures 5A and 5B show genes that were differentially expressed upon short and long-term rHuEpo treatments when compared to un-stimulated control cells. In the MCF-7 cell line (Figure 5A), *FOS* and *BCL2L1* were up-regulated and *JUN* was down-regulated after rHuEpo treatment independently of the treatment duration. *BCL2* and *CASP9* were up-regulated after short-term rHuEpo treatment, while long-term treatment down-regulated *BCL2* together with *BAD* and up-regulated *PMAIP1* and *NF-κβ*. In MDA-MB-231 cell line (Figure 5B) several genes were down-regulated after short-term treatment, namely *BAD*, *BAX*, *BBC3* and *PMAIP1*, while the expression of *BCL2L1* was increased. After long-term treatment, only *BAD* was deregulated; in contrast to short-term treatment, its increased expression was observed.

*rHuEpo and cDDP interaction.* Venn diagrams on Figures 5C and 5D show differentially expressed genes in cells that were exposed to cDDP in comparison to non-exposed control cells with respect to different rHuEpo treatments. In MCF-7 cells (Figure 5C), *BAX* and *BBC3* up-regulation was observed irrespectively of the rHuEpo treatment. *BCL2* was up-regulated in cells that were exposed to cDDP but were not treated with rHuEpo. Exposure of short-term rHuEpo treated cells to cDDP down-regulated several genes, namely *CASP9*, *PMAIP1*, *BCL2L1*, *NF-κβ* and *BCL2*, while *JUN* expression was increased. Long-term rHuEpo treated cells respond to cDDP exposure with *BAD* up-regulation. In MDA-MB-231 cell line (Figure 5D), *FOS*, *CASP9* and *CASP3* were up-regulated and *BCL2L1* was down-regulated after exposure to cDDP irrespectively of the rHuEpo treatment. In short-term rHuEpo treated MDA-MB-231 cells, exposure to cDDP increased the expression of *BAX* and *JUN. BAD* up-regulation was shown in short-term treated cells that were exposed to cDDP, while long-term rHuEpo treatment seems to antagonize its up-regulation.

### MAPK and PI-3K signalling pathways

**FIGURE 6:**
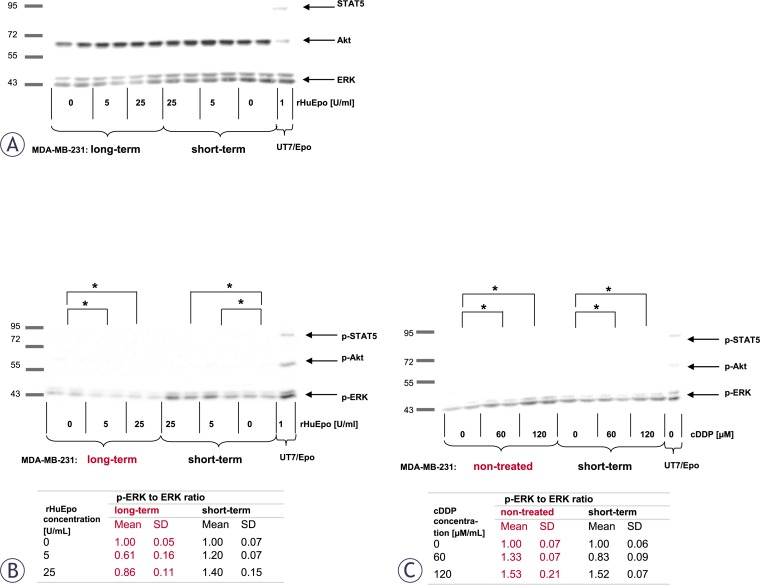
Involvement of MAPK (ERK), PI-3K (Akt) and Jak/STAT5 (STAT5) signaling pathways in Epo signaling for MDAMB-231 cell line. (A) Expression of ERK, Akt and STAT5 proteins in short and long-term rHuEpo treated cells. (B) ERK phosphorylation (p-ERK) in short and long-term rHuEpo treated MDA-MB-231cells. p-ERK to ERK ratios after rHuEpo treatment (5, 25 U/mL) were compared with non-treated samples. Table shows densitometry ratios and corresponding standard deviations (SD). (C) ERK phosphorylation (p-ERK) in short-term rHuEpo treated or non-treated cells after the exposure to cDDP. p-ERK to ERK ratios after exposure to cDDP (60, 120 μM/mL) were compared to samples that were not exposed to cDDP. Table shows densitometry ratios and corresponding standard deviations (SD). Asterisk (*) denotes statistical significant differences (p-ERK to ERK ratio) for Type I error α = 0.05. UT7/Epo cells were used as positive controls for Epo signaling.

In view of the evidence for the expression and functionality of EpoR in MCF-7 and MDA-MB-231 cells, we evaluated the ability of Epo to signal through well-established pathways that are thought to promote cell proliferation and cytoprotection, specifically the ERK, Akt and STAT5. The analysis of MDA-MB-231 cell line is presented on Figure 6. rHuEpo treatment or exposure to cDDP did not promote phosphorylation of ERK, Akt or STAT5 in MCF-7 cells (data not shown). We also confirmed that STAT5 is not expressed in MCF-7 cells, which was already reported by Yamashita *et al*.^46^ and is consistent with qPCR data from our laboratory (data not shown).

*rHuEpo effect*. We were able to detect a low level of ERK phosphorylation in short and long-term rHuEpo treated MDA-MB-231 cells (Figure 6B). Long-term treated cells became less responsive to the Epo stimulation in comparison to the control cells; there was also a statistically significant difference in ERK phosphorylation between short and long-term treated cells that was approximately 2.5-fold higher in short-term treated cells as measured by densitometry (Figure 6B). rHuEpo was not able to promote phosphorylation of Akt and no STAT5 expression was detected in MDA-MB-231 cells (Figure 6A), the observation that is in agreement with qPCR data from our laboratory (data not shown).

*rHuEpo and cDDP interaction*. Non-treated MDA-MB-231 cells that were exposed to cDDP for 4 h show an increase in ERK phosphorylation at both cDDP concentrations when compared to cells that were not exposed to cDDP (Figure 6C). After short-term rHuEpo treatment, 120 μM cDDP increased ERK phosphorylation, while 60 μM cDDP decreased the phosphorylation level in comparison with controls (Figure 6C). We could not detect any statistically significant change in the level of ERK phosphorylation after long-term rHuEpo treatment when compared to non-treated and short-term treated cells (data not shown).

## Discussion

Clinical trials with rHuEpo have shown decreased anaemia and improved quality-of-life for cancer patients receiving chemotherapy. In spite of these beneficial effects, rHuEpo was shown to cause detrimental effects on patient well-being, decreased loco-regional control of disease progression and decreased over-all survival.^15^–^18^ Mechanisms of the observed adverse clinical effects have remained elusive, but the most frequently considered hypothesis is the binding of cancer cell EpoR with exogenously administered rHuEpo. EpoR activation is considered to influence cancer cell growth in terms of stimulated proliferation, decreased apoptosis and increased resistance to therapy. It was reported that AP-1 (FOS and JUN) transcription factor is critical for the growth and proliferation of breast cancer cells^47^ and is also involved in the stimulation of NF-κβ transactivation activity.^48^ In erythroid cells, Epo was reported to co- or posttranslationally increase AP-1 activity.^49^ We therefore performed rHuEpo treatment of MCF-7 and MDA-MB-231 cells in order to assess the effect of rHuEpo treatment on cell proliferation and its potential to synergize with cDDP in suppression of breast cancer cell growth. We showed that MCF-7 and MDA-MB-231 cells express *EPOR* mRNA and on the basis of previous reports we consider the protein functional.^28^ The effect of time duration to rHuEpo exposure (long, short-term) was also addressed.

*rHuEpo effect.* The effect on MCF-7 cell proliferation and cytotoxicity seems to be influenced by the length of rHuEpo treatment. Clonogenic assays showed decreased number and size of colonies for short-term rHuEpo treated MCF-7 cells, while on the other hand colony number and size were increased with long-term stimulated cells (Figure 2). Surprisingly, rHuEpo in MCF-7 cells failed to elicit phosphorylation of ERK and Akt, therefore the activation of MAPK and PI-3K signalling cascades that are indicative for EpoR activation^6^,^7^ could not be confirmed in this treatment conditions. Results could also suggest that other signalling pathways may be involved. qPCR results (Figure 5) showed up-regulation of *BCL2* gene in short-term rHuEpo treated cells and down-regulation of this gene after long-term treatment, indicating involvement of *BCL2* in the proliferative and cytotoxic response to Epo. Additional up-regulation of *PMAIP1* gene was shown for long-term rHuEpo treated MCF-7 cells. Results suggest that in addition to genes involved in the cell sensitivity to apoptotic stimuli (*BCL2, PMAIP1*), Epo also modulates genes involved in cell proliferation (*FOS*, *NF-κβ*). Consequently, when growth conditions are near optimal, cells proliferate more rapidly, but as soon as an apoptotic stimulus is involved, cell survival is diminished. Using MDA-MB-231 cell line, no change in the proliferation level of rHuEpo treated cells was observed despite low level of ERK phosphorylation. Signalling through the growth factor receptor tyrosine kinase pathway in PR(−) tumours was indicated previously.^28^ ERK phosphorylation was significantly reduced after long-term treatment, indicating that cells are non-responsive to Epo stimulation, probably due to saturation of MAPK signalling pathway or regulation of cytosolic phospholipase A2.^50^

Furthermore, qPCR data show that short-term rHuEpo up-regulates *BCL2L1* and down-regulates *BAD*, *BAX*, *PMAIP1* and *BBC3*. In line with this, rHuEpo treatment did not trigger Akt phosphorylation which was previously shown to act as an activator of apoptotic process.^51^,^52^ Comparable proliferation rate and survival of rHuEpo treated and control MDA-MB-231 cells and the presence of activated ERK in all treatment groups may indicate, that lack of rHuEpo effect on the proliferation of MDA-MB-231 cells is due to auto-activating mutations or alteration in gene expression that result in constitutive activation of signalling pathways that drive proliferation at the nearly maximal rate.^53^

*rHuEpo and cDDP interaction.* Given the controversy of reports explaining Epo role in the cytoprotection of cancer cells,^31^,^34^,^54^ we addressed effects of rHuEpo treatment and exposure to cDDP and their potential interaction. p53 modulates cell response to cDDP by transcriptional activation of *BAX*, *PMAIP1* and *BBC3* and consequent suppression of *BCL2* expression.^55^ Therefore we investigated, whether difference in *p53* status could influence cell response to rHuEpo and cDDP. Using both types of survival assays, we detected attenuated anti-proliferative, apoptotic or senescence-promoting effects of cDDP with short-term rHuEpo treated MCF-7 cells (Figure 3A and 4A). On the other hand, survival of long-term rHuEpo treated cells was significantly lower after the exposure to cDDP (Figure 3B and 4B), particularly at higher cDDP concentrations. rHuEpo pretreatment seems to render MCF-7 cells to be more sensitive to the cytotoxic effect of cDDP. Together with this, qPCR analysis exposed differential gene expression for short and long-term rHuEpo effects (Figure 5C). Results therefore suggest that MCF-7 cell response to cDDP depends on the length of rHuEpo exposure. Furthermore, no significant change in ERK and Akt activation was shown after cDDP treatment which is crucial for the induction of apoptosis.^51^,^52^ These results indicate that Epo may modulate cell response to cDDP through deregulation of ERK and Akt expression. In MDA-MB-231 cell line, clonogenic assays, but not colorimetric ones (Figure 3C and 4C), suggest poorer cell proliferation and survival for short-term treated cells and are in agreement with qPCR results which exposed promoted anti-survival genotype that was evident from the up-regulation of several pro-apoptotic genes (Figure 5D). Clonogenic assays may be more informative because they measure cell number together with cell capacity to form colonies over longer periods of time, while on the other hand colorimetric assays are short-term and only measure cell activity of superoxide dismutase (SOD). Surprisingly, we could not confirm any effect of long-term rHuEpo exposure on the level of proliferation, clonogenicity, qPCR or western blot in this cell line (Figure 3D, 4D, 5D and 6). MDAMB-231 cells with the mutated *p53* have disrupted apoptotic machinery that could aid to cell un-responsiveness to cytoprotective and proliferative effect of rHuEpo The lack of cell response could also be explained in terms of more aggressive phenotypes for ER(+)/PR(−) tumours^29^ and the postulated correlations to the expression of EpoR and steroid receptors.^30^,^56^

## Conclusions

Our study showed that Epo has a contrasting action in breast cancer biology that depends on the duration of exposure to rHuEpo, presence of cytotoxic stimuli, ER/PR and *p53* status. The correlation between ER/PR and Epo was shown previously.^30^,^56^–^58^ Our study indicates that besides ER/PR status, also p53 is involved in Epo induced tumour response.

Proliferation and survival characteristics of MCF-7, cells with ER(+)/PR(+) status and wild type *p53,* are opposite during short or long term rHuEpo exposure. Prolonged exposure to rHuEpo drives MCF-7 cells to increased proliferation and induced sensitivity to cDDP. In contrast, MDA-MB-231 cells with ER(+)/PR(−) status and mutated *p53* are almost irresponsive to rHuEpo exposure. Functional p53 and ER(+)/PR(+) status seems to be crucial for long-term rHuEpo driven modification of cancer cells. Interestingly, in MCF-7 *in vitro* assay (with optimal growth conditions) prolonged rHuEpo exposure during cDDP chemotherapy is beneficial, while rHuEpo treatment alone is not. These results have to be verified in a setup representing *in vivo* conditions (tumour hypoxia, treatment regime, …).

Furthermore, Epo is involved in transcription regulation of *BAX*, *PMAIP1*, *BBC3* and *BCL2*, results suggesting its involvement in p53-modulated cell response to cDDP. Epo also modulates the expression of *NF-κβ*, *FOS* and *JUN* transcription factors and in MDA-MB-231 cells reduces MAPK kinase signal transduction.

To conclude, ER/PR and p53 genetic signature may be used to predict the beneficial or maleficent effect of rHuEpo supportive therapy in the individual patient. Whole-genome expression studies need to be employed in order to identify the main components of Epo/EpoR signal transduction that modulate cell proliferation and cell sensitivity to cytotoxic stimuli.

## Figures and Tables

**TABLE 1:** Details on genes of interest and reference genes

**Genes of interest, GOI**					

**Gene symbol**	**Gene Name**	**Primer sequence**	**Ref.seq**	**Amplicon length**	**Primer Eff**
*FOS*	FBJ murine osteosarcoma viral oncogene homolog	Fw: 5′-CTACCACTCACCCGCAGACT-3′ Rev: 5′-AGGTCCGTGCAGAAGTCCT-3′	NM_005252.2	72	2
*JUN*	jun-proto oncogene	Fw: 5′-CCAAAGGATAGTGCGATGTTT-3′ Rev: 5′-CTGTCCCTCTCCACTGCAAC-3′	NM_002228.2	62	2
*NFκβ*	nuclear factor of kappa light polypeptide gene enhancer in B-cells 1	Fw: 5′-GGTGCCTCTAGTGAAAAGAACAAGA-3′ Rev: 5′-GCTGGTCCCACATAGTTGCA-3′	NM_003998.3	68	1.722
*TP53*	tumor protein p53	Fw: 5′-CCCCAGCCAAAGAAGAAAC-3′ Rev: 5′-AACATCTCGAAGCGCTCAC-3′	NM_000546.4	77	1.922
*BAD*	BCL2-assocciated agonist of cell death	Fw: 5′-GAGTGACGAGTTTGTGGACTCCTT-3′ Rev: 5′-TGTGCCCGCGCTCTTC-3′	NM_004322.2	61	2.055
*BAX*	BCL2-assocciated X protein	Fw: 5′-ATGTTTTCTGACGGCAACTTC-3′ Rev: 5′-ATCAGTTCCGGCACCTTG-3′	NM_004324.3	104	1.812
*BBC3*	BCL2 binding component 3 [PUMA]	Fw: 5′-GACCTCAACGCACAGTACGA-3′ Rev: 5′-GAGATTGTACAGGACCCTCCA-3′	NM_001127240.1	84	1.651
*BCL2*	B-cell CLL/lymphoma 2	Fw: 5′-TCCCTCGCTGCACAAATACTC-3′ Rev: 5′-ACGACCCGATGGCCATAGA-3′	NM_000633.2	72	2.117
*BCL2L1*	BCL2-like 1 [BCL-XL]	Fw: 5′-CTTTTGTGGAACTCTATGGGAACA-3′ Rev: 5′-CAGCGGTTGAAGCGTTCCT-3′	NM_138639.1	70	2.023
*CASP3*	caspase 3, apoptosis-related cysteine peptidase	Fw: 5′-GCCTACAGCCCATTTCTCCAT-3′ Rev: 5′-GCGCCCTGGCAGCAT-3′	NM_004346.3	57	2.025
*CASP9*	caspase 9, apoptosis-related cysteine peptidase	Fw: 5′-GGAAGCCCAAGCTCTTTTTC-3′ Rev: 5′-AAGTGGAGGCCACCTCAAA-3′	NM_001229.2	75	1.997
*PMAIP1*	phorbol-12-myristate-13-acetate-induced protein 1 [NOXA]	Fw: 5′-GGAGATGCCTGGGAAGAAG-3′ Rev: 5′-CCTGAGTTGAGTAGCACACTCG-3′	NM_021127.2	94	2.086
*EPOR*	erythropoietin receptor	Fw: 5′-TTGGAGGACTTGGTGTGTTTC-3′ Rev: 5′-AGCTTCCATGGCTCATCCT-3′	NM_000121.2	101	1.813

**Reference genes**					

**Rplp0**	ribosomal protein, large, P0	Fw: 5′-TCTACAACCCTGAAGTGCTTGAT-3′ Rev: 5′-CAATCTGCAGACAGACACTGG-3′	NM_001002.3	96	2.073
**GAPDH**	glyceraldehyde-3-phosphate dehydrogenase	Fw: 5′-AGCCACATCGCTCAGACAC-3′ Rev: 5′-GCCCAATACGACCAAATCC-3′	NM_002046.3	66	1.999
*PpiA*	peptidylprolyl isomerase A (cyclophilin A)	Fw: 5′-ATGCTGGACCCAACACAAAT-3′ Rev: 5′-TCTTTCACTTTGCCAAACACC-3′	NM_021130.3	97	1.981
*YWHAZ*	tyrosine 3 – monooxygenase/tryptophan 5 –monooxygenase activation protein	Fw: 5′-GATCCCCAATGCTTCACAAG-3′ Rev: 5′-TGCTTGTTGTGACTGATCGAC-3′	NM_003406.2	130	1.833
*HPRT1*	hypoxanthine phosphoribosyltransferase 1	Fw: 5′-TGACCTTGATTTATTTTGCATACC-3′ Rev: 5′-CGAGCAAGACGTTCAGTCCT-3′	NM_000194.2	102	2.013
*18S*	18S ribosomal RNA	Fw: 5′-GGAGAGGGAGCCTGAGAAAC-3′ Rev: 5′-TCGGGAGTGGGTAATTTGC-3′	NR_003286.2	70	1.999
*ACTB*	actin, beta	Fw: 5′-CCAACCGCGAGAAGATGA-3′ Rev: 5′-CCAGAGGCGTACAGGGATAG-3′	NM_001101.3	97	1.938
